# Thrombocytopenia and absent radii (TAR) syndrome associated with bilateral congenital cataract: a case report

**DOI:** 10.1186/1752-1947-6-168

**Published:** 2012-06-28

**Authors:** Ahmed Omran, Shaimaa Sahmoud, Jing Peng, Usman Ashhab, Fei Yin

**Affiliations:** 1 Department of Pediatrics, Xiangya Hospital of Central South University, No. 87 Xiangya Road, Changsha, Hunan, 410008, China; 2 Pediatrics and neonatology department, Suez Canal University, Ismailia, Egypt

## Abstract

**Introduction:**

Thrombocytopenia with absent radii is a rare congenital defect with hypomegakaryocytic thrombocytopenia and bilateral radial aplasia that may have additional anomalies. We report the case of a girl baby with thrombocytopenia and absent radii syndrome and bilateral congenital cataract. This anomaly has not been previously reported in the children of a non- consanguineous marriage.

**Case presentation:**

This case report describes a two-day-old girl baby of Arab origin with thrombocytopenia and absent radii syndrome and bilateral congenital cataract.

**Conclusions:**

This report describes a finding of bilateral congenital cataract associated with thrombocytopenia and absent radii syndrome that has been reported only once before in the literature. This case report highlights a new ocular manifestation in one of the bone marrow failure syndromes.

## Introduction

Thrombocytopenia with absent radii (TAR) is a rare congenital defect with hypomegakaryocytic thrombocytopenia and bilateral radial aplasia. Although first described in 1929, it was defined as a syndrome by Hall *et al.* in 1969 [1]. It was initially thought to be a variant of Fanconi's anemia but is now known to be a separate entity and some reports suggest it is more common [2]. The two features that are currently essential for the definition of the syndrome are hypomegakaryocytic thrombocytopenia and bilateral radial aplasia; other characteristics include other limb abnormalities, as well as intermittent leukocytosis, eosinophilia, anemia secondary to hemorrhage, cardiac defects, renal anomalies, mental retardation, and milk protein allergy [3]. This report describes a baby girl with TAR syndrome in association with bilateral congenital cataract which has been reported only once before in the literature.

## Case presentation

A two-day-old term baby girl of Arab origin was the first child for young non-consanguineous parents. The pregnancy was uneventful without any history of maternal illness, fever or rash. The baby was delivered by normal spontaneous vaginal delivery outside our hospital. The family pedigree is negative for congenital malformation.

**Figure 1:**
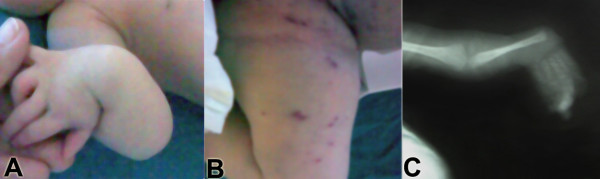
**Clinical signs and X-ray findings in the baby girl.** (**A**) Radial deviation of the right upper limb with no thumb deformity. (**B**) Multiple purpuric eruptions in the anterior aspect of the right lower limb. (**C**) X-ray of the left upper limb showing absent radius.

The baby was referred to the neonatal intensive care unit of the Suez Canal University Hospital, Egypt, when she was two days old due to malformed upper extremities, multiple purpuric eruptions and bilateral corneal opacities (Figures 1 A, B).

Physical examination at presentation revealed a baby with stable vital signs who was alert, pink and active, with a length of 49 cm, a weight of 2,800 g, and a head circumference of 34 cm (all on the 50th percentile), bilateral corneal opacities without any facial dysmorphic features, multiple purpuric eruptions mostly on the anterior aspect of the lower limbs and abdomen. She had bilaterally absent radii and radial deviation of both upper limbs with no thumb deformity. There were no deformities in the lower limbs. Abdominal examination revealed no organomegaly, there was no clinical evidence of congenital heart disease and the other systems were normal.

Initial laboratory investigations gave the following results: complete blood count: hemoglobin, 10 g/dL; mean corpuscular volume, 105 fl; mean corpuscular hemoglobin, 34 pg; reticulocytic count, 6%; TLC: 49,000/uL, 60% s. neutrophils, 5% myelocytes, 5% metamyelocyte, 10% normoblasts; platelet count, 20,000/uL; prothrombin time, 12.8 seconds (cont 12); partial thromboplastin time, 28.0 seconds (cont 28). Liver function tests had the following results: bilirubin (total), 6.14 mg/dL; (direct), 0.24 mg/dL; glutamic pyruvic transaminase, 28 U/L; and glutamic oxaloacetic transaminase, 34 U/L. Electrolytes and kidney function were: Na, 136 mmol/L; K, 4.8 mmol/L; serum creatinine, 0.9 mg/dL. TORCH screens were normal with rubella IgG antibodies, 12 Eu (0–20) and Rubella IgM antibodies, 0.20 Eu (0–40). Radiography of the forearm showed bilateral absence of radii (Figure 1 C). Her abdominal and cranial ultrasonography were normal. Based on these physical findings, thrombocytopenia, and X-ray results, the diagnosis of TAR syndrome was made. At this time she received two platelet transfusions and had a post transfusion platelet count of 55,000/uL. Bone marrow aspiration performed when she was seven days old revealed previously undetected megakaryocytes in the examined smears. It also showed that the myeloid cell line was mildly hyperactive with preservation of normal sequential maturation, the myeloid/erythroid ratio was slightly decreased, the erythroid cell line had mild hyperplasia with dysplastic changes and an increase in the early stages. Ophthalmology consultation revealed irregular red reflex with bilateral nuclear congenital cataract, both eyes were equal and normal in size.

She is now on prophylactic platelet transfusion twice weekly and is scheduled for cataract surgery by the age of six weeks to prevent irreversible amblyopia and sensory nystagmus, starting with the right eye as it is denser. The parents refused to do genetic studies for their baby.

## Discussion

TAR syndrome is a rare genetic disorder that may be associated with multiple additional anomalies. Thrombocytopenia, which may be transient, is seen in 100% of cases diagnosed with TAR syndrome [1]. Patients are usually diagnosed at birth, due to thrombocytopenia as they present with petechial rash or overt hemorrhage such as bloody diarrhea in the first week of life or later during the next four months. Platelet counts at birth are usually 15,000 to 30,000/uL [3,4]. The exact pathophysiology of the thrombocytopenia is still unclear, but it may be explained by the following different mechanisms: (1) the absence of humoral or cellular stimulators of megakaryocytopoiesis, (2) the absence of megakaryocytic progenitor cells, (3) cellular defects in megakaryocytic precursors (for example, receptor defects) or (4) the presence of humoral or cellular inhibitors of megakaryocytopoiesis [5].

Elevated white blood cells have also been reported in other TAR syndrome case reports [6]. Megakaryocytes are absent in two thirds of the bone marrows aspirated and the rest are decreased in number and are small, immature and vacuolated [7]. The prognosis in TAR is better than in any of the other inherited bone marrow failure syndromes.

The upper limb abnormalities range from isolated absent radii with normal thumbs, as is demonstrated in our case, to phocomelia [5]. The etiology of radial aplasia in TAR syndrome is a primary failure of chondrogenesis and not a disorder of vasculogenesis as in other disorders with absent radii [6]. Lower extremity anomalies occur in 46% of patients but are usually less severe than those of the upper limbs. These abnormalities include hip dislocation, femoral torsion, tibia torsion valgus and varus deformity and deformity of the knee [5]. None of these were seen in our case.

In an attempt to understand the genetic basis of TAR syndrome, Klopocki *et al.* reported that TAR syndrome has a complex pattern of inheritance associated with a common interstitial microdeletion of 200 kb on chromosome 1q21.1 and an additional, as yet unknown, modifier [8]. Houeijeh *et al.* mentioned that the identification of the 1q21.1 deletion allows for confirmation of the TAR syndrome diagnosis, particularly in patients with atypical phenotypes, and it also allows for accurate genetic counseling, especially when it occurs *de novo*[9].

The genomic structure of the 1q21.1 breakpoint regions is extremely complex, with at least four large segmental duplication blocks ranging in size from 270 kb to 2.2 Mb. Within 1q21.1 there are two areas where a deletion can be found: the TAR area for the TAR syndrome and the distal area for other anomalies. The 1q21.1 deletion syndrome will commonly be found in the distal area, but an overlap with the TAR area is possible [10].

This case report describes an interesting finding of bilateral congenital cataract in association with the TAR syndrome. This ocular anomaly has been reported once before in association with the TAR syndrome but in a consanguineous Mayan girl, who had, in addition to cataract, other ocular anomalies including glaucoma, megalocornea and blue sclera [11].

Eye abnormalities are seen in 26% of individuals with 1q21.1 microdeletion and may include strabismus, chorioretinal and iris colobomas, microphthalmia, hypermetropia, Duane anomaly, and congenital cataract [12].

The human gene encoding the lens intrinsic membrane protein MP70 that makes the majority of the transmembrane protein found in fiber cells of the vertebrate ocular lens was regionally mapped to q21.1 on the long arm of chromosome 1. These data support the hypothesis that a genetic lesion in the gene encoding the lens intrinsic membrane protein MP70 may be the underlying molecular defect for some forms of human hereditary cataract [13].

Congenital cataract has been also reported with other congenital infection syndromes and bone marrow failure syndromes [14,15].

## Conclusions

In this case report we present an unusual ocular manifestation in a baby girl with TAR syndrome. This is the first report of bilateral congenital cataract in association with TAR syndrome in a non-consanguineous Arabic baby girl. This case may add a new finding to the ocular manifestations in one of the bone marrow failure syndromes.

## Consent

Written informed consent was obtained from the patient’s next-of-kin for publication of this case report and any accompanying images. A copy of the written consent is available for review by the Editor-in-Chief of this journal.

## Competing interests

The authors declare that they have no competing interests.

## Authors’ contributions

AO provided care during the patient’s admission, drafted the manuscript, carried out the literature search and prepared the illustrations. SS provided care during the patient’s admission and shared in preparation of the illustrations. JP and UA shared in manuscript preparation. FY was responsible for the final proof reading of the manuscript. All authors read and approved the final manuscript.

## Authors’ information

AO is a PhD student, Pediatrics Department, Central South University, China and lecturer assistant of pediatrics, Faculty of Medicine, Suez Canal University, Egypt. SS is an MD student and lecturer assistant of pediatrics, Faculty of Medicine, Suez Canal University, Egypt. JP is an Attending Physician of Pediatrics, Central South University, China. UA is a master degree student, Pediatrics Department, Central South University, China. FY is a Professor and head of the Pediatrics Department, Central South University, China.
